# Cytotoxic Potential of *Diospyros villosa* Leaves and Stem Bark Extracts and Their Silver Nanoparticles

**DOI:** 10.3390/plants12040769

**Published:** 2023-02-08

**Authors:** Oluwatosin Temilade Adu, Yougasphree Naidoo, Johnson Lin, Depika Dwarka, John Mellem, Hosakatte Niranjana Murthy, Yaser Hassan Dewir

**Affiliations:** 1 Department of Biological Sciences, School of Life Sciences, College of Agriculture, Engineering and Science, University of KwaZulu-Natal, Westville Campus, Private Bag X54001, Durban 4000, South Africa; 2 Department of Microbiology, School of Life Sciences, College of Agriculture, Engineering and Science, University of KwaZulu-Natal, Private Bag X54001, Durban 4000, South Africa; 3 Department of Biotechnology and Food Technology, Durban University of Technology, P.O. Box 1334, Durban 4000, South Africa; 4 Department of Horticultural Science, Chungbuk National University, Cheongju 28644, Republic of Korea; 5 Plant Production Department, College of Food & Agriculture Sciences, King Saud University, Riyadh 11451, Saudi Arabia

**Keywords:** cell viability, cytotoxicity, camptothecin, *Diospyros villosa*, nanoparticles

## Abstract

*Diospyros villosa* is traditionally used for an anti-bacterial property. Its cytotoxic effects have not been studied before. Therefore, this study aimed to examine the nutritional properties as well the cytotoxic effects of *D. villosa*. The leaves and stem barks were subjected to three different extraction methods (methanol, chloroform and hexane) and their nanoparticles were synthesized at two different temperatures (room temperature and at 80 °C). Thereafter, extracts were assessed using the associated AOCC protocols, for their nutritional content (moisture, fibre, proteins, lipid, ash and hydrolysable carbohydrates). *Diospyros villosa* extracts and their corresponding nanoparticles were then incubated overnight with cancerous and noncancerous cell lines to evaluate their cytotoxic potential. The nutritional analysis revealed that both young and mature leaves were rich sources of protein having values of 14.95% and 11.37% respectively. The moisture content was observed to be higher in all the leaf types (8.54 ± 0.75%, 9.67 ± 0.98% and 7.40 ± 0.80%) compared to the stem (2.13 ± 0.07%) respectively. The MTT cytotoxicity assay showed that the cell viability of MCF-7 cell lines was significantly lower when exposed to hexane and chloroform leaves extracts of *D. villosa* (IC_50_ of 26.64 and 26.07 µg mL^−1^) respectively, compared to camptothecin (36.54 µg mL^−1^). Similarly, the MCF-7 cell viability was observed to be significantly lower when exposed to hexane and chloroform stem extracts of *D. villosa* (IC_50_ of 24.57 and 3.92 µg mL^−1^), compared to camptothecin (36.54 µg mL^−1^). The cell viability of A549 cell lines was also found lower when exposed to the hexane and chloroform extracts (IC_50_ of 7.76 and 4.59 µg mL^−1^) compared to camptothecin (IC_50_ of 19.26 µg mL^−1^). Furthermore, the viability of A549 cell lines was found lower when exposed to hexane and chloroform stem extracts of *D. villosa* (IC_50_ of 10.67 and 5.35 µg mL^−1^) compared to camptothecin (19.26 µg mL^−1^). The biosynthesized nanoparticles further displayed an anticancer activity with an IC_50_ value of 4.08 µg mL^−1^ when compared to the control (36.54 µg mL^−1^). However, the HEK293 cell viability was observed to be significantly higher on exposure to hexane stem extracts of *D. villosa* (IC_50_ of 158.5 µg mL^−1^) compared to camptothecin (IC_50_ of 14.77 µg mL^−1^). Therefore, *Diospyros villosa* leaves, stem bark and nanoparticles synthesized showed high potential for being considered as a candidate for an anti-cancer regimen.

## 1. Introduction

Cancer is a complex disease of uncontrolled growth of tumour cells due to signalling failure of oncogenic expressions resulting in many different types of cancers based on the origin of tumours in the particular organs [1]. An estimated 19.3 million new cancer cases and almost 10 million cancer deaths were reported in 2020 [2]. In fact, almost 11.7% of all the new cancer cases were reported to be female breast cancer and identified to be the most commonly diagnosed cancer in women [3]. Up to now, efforts are made to develop efficient approaches not only to diagnose cancer but also to treat the disease. A variety of therapeutic approaches including chemotherapy [4], molecularly targeted therapy [5], gene therapy [6], radiotherapy [7], immunotherapy [8] phototherapy [9], and embolotherapy [10] have been extensively applied to treat cancers in clinic. All these therapeutic measures present severe effects on the patients. However, there is still a need to secure a more reliable, cheaper and readily available therapeutic measure, with limited side effects.

There are many medicinal plants with therapeutic properties that have been used traditionally in many countries and are also being researched by various groups in the form of extracts against different types of cancer for possible treatments [11,12,13]. Additionally, dietary supplementation of phytonutrients is an emerging trend that provides a multifaceted defensive mode against various maladies such as cancer by limiting tumour development by binding to the cancer cell membrane or their receptors, thereby initiating cytotoxicity and apoptosis inhibiting tumour growth [14]. These phytonutrients possess certain key advantages over alternative chemotherapy agents such as their affinity and level of tissue penetration, strong target specificity and low toxicity [15] Medicinal plant therapeutic agents also contribute indirectly by activating the endogenous defence systems by modulating cellular signalling processes [16] and thereby enhancing the overall health status. There is an abundance of medicinal shrubs, vegetables and trees in South Africa that are yet to be prodded meticulously for their health-promoting properties. Similarly, nanoparticles synthesized from green plants were reported to possess unique biological properties and hence become useful in therapeutics and drug delivery [17]. The biosynthesized nanoparticles eradicate cancer cells by flow and penetration to different regions of tumours through blood vessels into the target cells [18].

*Diospyros villosa* (L.) De Winter (*D. villosa*) is an African plant which naturally occurs in southern parts of the continent. *D. villosa* root was reported to be used by a group of herbalists found in the botanically diverse Western Cape of South Africa to treat gastrointestinal complaints, worms, and flatulence [19]. Also, the root of the *D. villosa* plant was used in the rural community of northern Maputaland to treat pain and dysmenorrhea [20]. *Diospyros ferrea* (Wild.) leaves nanoparticles were reported for their anti-cancer activities against MCF-7 cancer cell lines [21]. Hence, this research study was then geared towards making a significant contribution to the present search being carried out to ascertain the nutritive contents of *D. villosa* leaves and stem bark and to investigate the anti-cancer properties of *D. villosa* leaves and stem bark as well as its nanoparticles on breast cancer cell lines (MCF-7), human embryonic kidney immortalized cell lines (HEK 293), and adenocarcinomic human alveolar basal epithelial cancer cells (A549).

## 2. Results

The yield of different extracts of *D. villosa* leaves and stem bark is given in Table 1. It was observed that methanol extraction in the leaves produced a maximum yield of 10.8%, whereas chloroform and hexane extraction in the leaves yielded 8.4% and 7.1% respectively. Similarly, the methanol extraction in the stem bark produced a yield of 7.2% meanwhile, chloroform and hexane extraction yielded 7.9% and 10.3% respectively. The yield obtained from leaves nanoparticles at room temperature and 80 °C was observed to be 7.4% and 5.5% respectively. Also, the percentage yield from stem nanoparticles at room temperature and 80 °C was found to be 4.0% and 3.95% respectively.

The proximate analysis of different extracts of *D. villosa* leaves and stem bark is given in Table 2. It was observed that the protein content of mature leaves of *D. villosa* was found significantly (*p* < 0.05) higher compared to the stem F_(3, 11)_ = 51.45, *p* = 0.0009. Similarly, the moisture content of the leaves (emergent, young and adult) was found significantly (*p* < 0.05) higher compared to the stem. Also, the moisture content in the leaves was found significantly higher compared to the stem bark. Meanwhile, the moisture content was not as much as the reported value (14.83%) in the leaves of *Diospyros mespiliformis*-a member of the same family (*Ebenaceae*) by Ebbo et al. [22]. The protein content in the mature leaves (14.95%) was slightly higher than the reported values (11.49%) [22]. Furthermore, the crude fibre content in the leaves and stem bark was higher compared to the reported value in *Diospyros mespiliformis* leaves.

The MTT assay was used to determine the cytotoxicity of the *D. villosa* leaves and stem using different extraction media as well as the biosynthesized AgNPs at different temperatures (RT and 80 °C) on cancerous and non-cancerous cell lines. For the extract to be anticancer, it should display toxicity on MCF-7, or A549 cancer cells and mild reactivity to HEK293 with further supporting evidence of IC_50_. The lower the IC_50_ values indicated, the higher the cytotoxic activity in cancerous cells.

**Figure 1:**
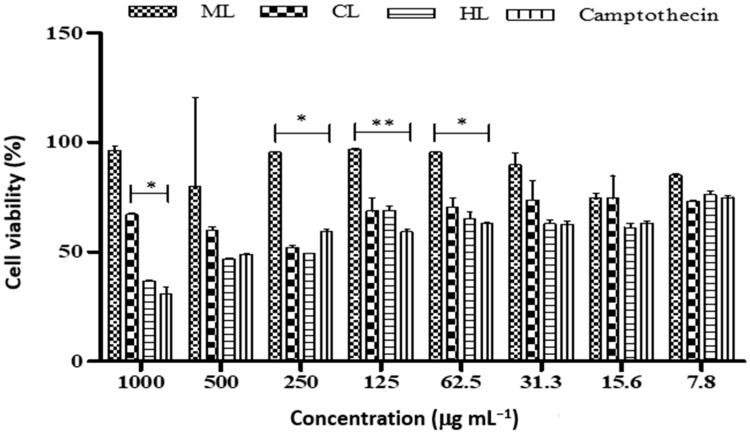
Cell viability of MCF-7 cancer cell line treated with different concentrations of *D. villosa* leaves. * (Camptothecin vs. CL); (Camptothecin vs. ML); *p* < 0.05. ** (Camptothecin vs. ML), *p* < 0.01.

Among the different leaf extracts, hexane extract showed a noteworthy cytotoxic effect on the MCF-7 cell line (IC_50_ 26.64 µg mL^−1^) and chloroform extract showed a significant cytotoxic effect (IC_50_ 26.07 µg mL^−1^) (Table 3 and Figure 1). However, the methanolic leaf extract showed the best cytotoxic effect (IC_50_ 7.09 µg mL^−1^) in MCF-7 cells. The hexane, chloroform and methanol leaf extract demonstrated greater anti-cancer activity than the standard camptothecin (IC_50_ 36.54 µg mL^−1^).

**Figure 2:**
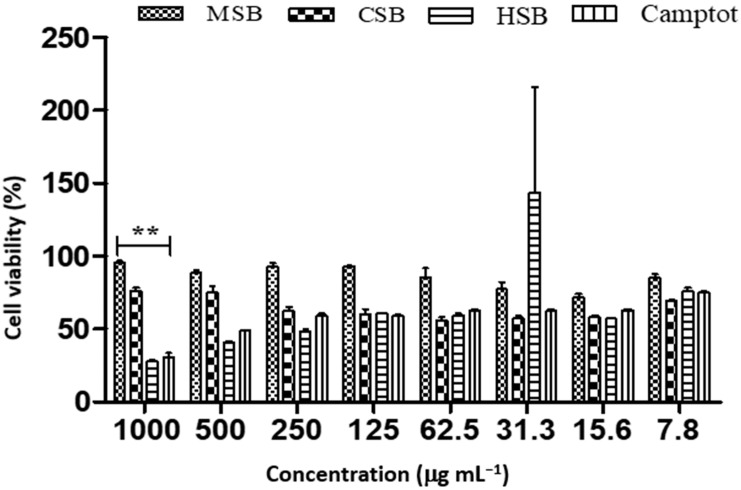
Cell viability of MCF-7 cancer cell lines treated with different concentrations of *D. villosa* stem extract. ** (Camptothecin vs. MSB), *p* < 0.01.

The cytotoxicity of MCF-7 cells by both hexane (IC_50_ 24.57 µg mL^−1^) and chloroform stem extracts (IC_50_ 3.919 µg mL^−1^) (Table 4 and Figure 2) was much greater compared to camptothecin (IC_50_ value of 36.54 µg mL^−1^). However, the greatest anticancer activity produced in MCF-7 cells was demonstrated by the methanolic stem extract (IC_50_ 0.17 µg mL^−1^).

**Figure 3:**
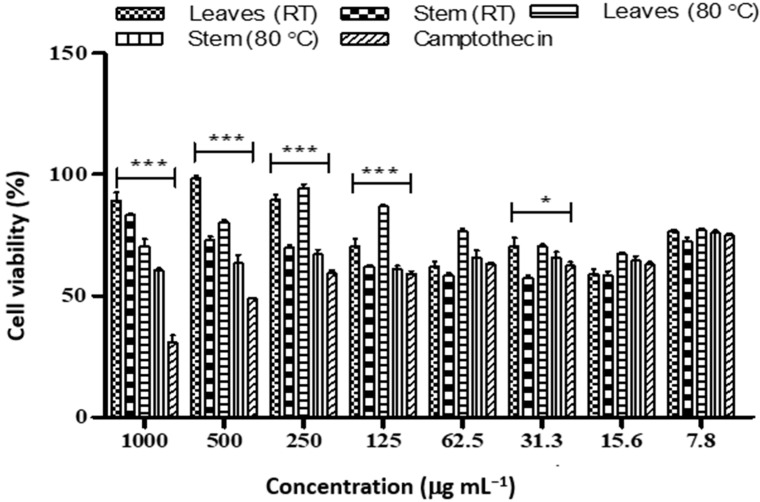
Cell viability of MCF-7 cancer cell lines treated with different concentrations of nanoparticles synthesized from *D. villosa* leaves and stem extract at room temperature and 80 °C. * (ML vs. Camptothecin), *p* < 0.05; *** (ML vs. Camptothecin), *p* < 0.001.

The *Diospyros villosa* stem nanoparticles biosynthesized at RT showed a significant toxic effect on MCF-7 (4.08 µg mL^−1^) compared to Camptothecin (36.54 µg mL^−1^) (Table 5 and Figure 3). In addition, the leaves nanoparticle synthesized at RT showed a significant toxic effect on MCF-7 cell lines (IC_50_ 2.03 µg mL^−1^) The IC_50_ of the leaves and stem nanoparticles synthesized at 80 °C was found to be 2.53 and 5.11 µg mL^−1^ respectively.

**Figure 4:**
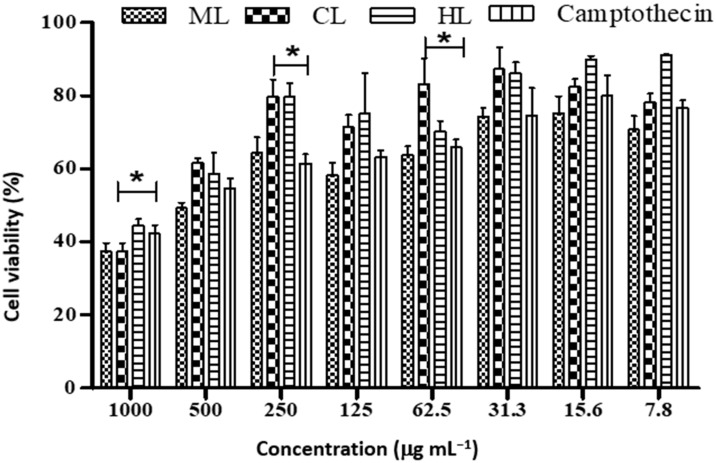
Cell viability of HEK293 cancer cell lines treated with different concentrations of *D. villosa* leaves extract. * (ML vs. Camptothecin); * (CL vs. camptothecin), *p* < 0.05.

The viability of the HEK 293 cell line was observed to be higher on exposure to methanolic leaves extract of *D. villosa* (IC_50_ 41.85 µg mL^−1^), chloroform leaves extract (IC_50_ of 198.5 µg mL^−1^) and hexane leaf extract (IC_50_ of 158.5 µg mL^−1^) compared to camptothecin (IC_50_ of 14.77 µg mL^−1^) (Table 6 and Figure 4).

**Figure 5:**
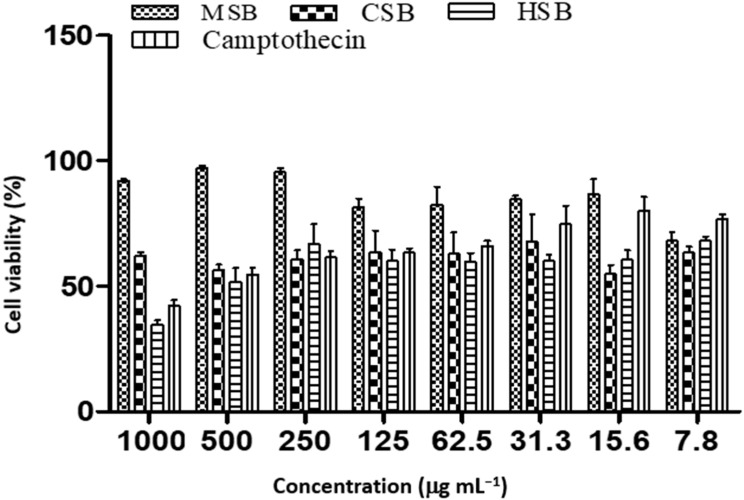
Cell viability of HEK293 cancer cell lines treated with different concentrations of *D. villosa* stem extract.

The viability of HEK293 cells was further observed to be greater when exposed to hexane stem extract of *D. villosa* (IC_50_ of 45.13 µg mL^−1^) (Figure 5). However, the viability of the HEK293 cells was largely affected when treated with the chloroform (IC_50_ of 3.93 µg mL^−1^) and methanolic (IC_50_ of 0.10 µg mL^−1^) extracts of *D. villosa* (Figure 5). Camptothecin produced an IC_50_ of 14.77 µg mL^−1^ (Table 7).

**Figure 6:**
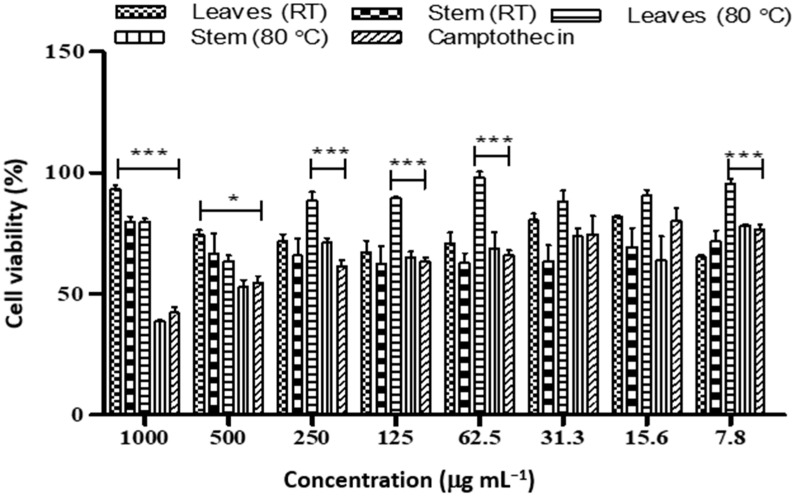
Cell viability of HEK 293 cancer cell lines treated with different concentrations of nanoparticles synthesized from *D. villosa* leaves and stem extract at room temperature and 80 °C. * (Leaves RT vs. Camptothecin); *p* < 0.05. *** (Leaves RT vs. Camptothecin), *p* < 0.001; *** (Leaves 80 °C vs. Camptothecin), *p* < 0.001.

*Diospyros villosa* leaves nanoparticles biosynthesized at RT showed a significant toxic effect on the HEK293 cell line (IC_50_ of 4.77 and 7.09 µg mL^−1^) compared to camptothecin (14.77 µg mL^−1^) (Table 8 and Figure 6). The viability of HEK293 cells was higher when exposed to leaves and stem nanoparticles (IC_50_ of 333.8 and 51.36 µg mL^−1^) of *D. villosa* (synthesized at 80 °C).

**Figure 7:**
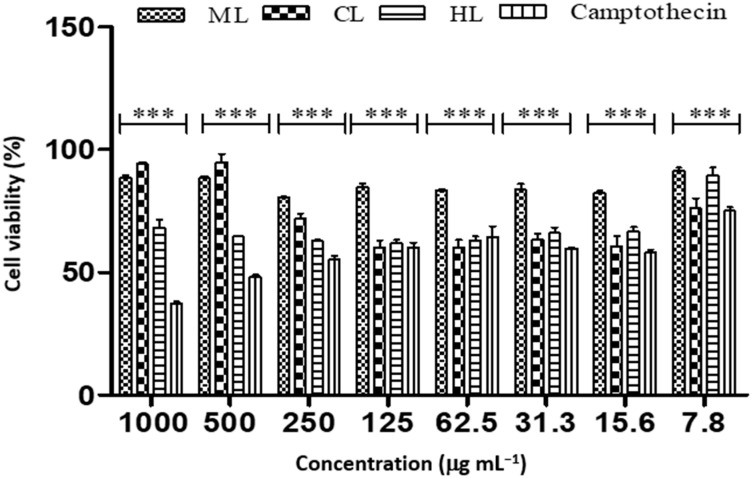
Cell viability of A549 cancer cell lines treated with different concentrations of *D. villosa* leaves extract. *** (ML vs. Camptothecin), *p* < 0.001.

The cell viability of A549 cells on exposure to chloroform (IC_50_ of 4.592 µg mL^−1^) and hexane leaves extract (IC_50_ of 7.76 µg mL^−1^) showed a greater anti-cancer effect compared to camptothecin (IC_50_ of 19.26 µg mL^−1^) (Table 9 and Figure 7).

**Figure 8:**
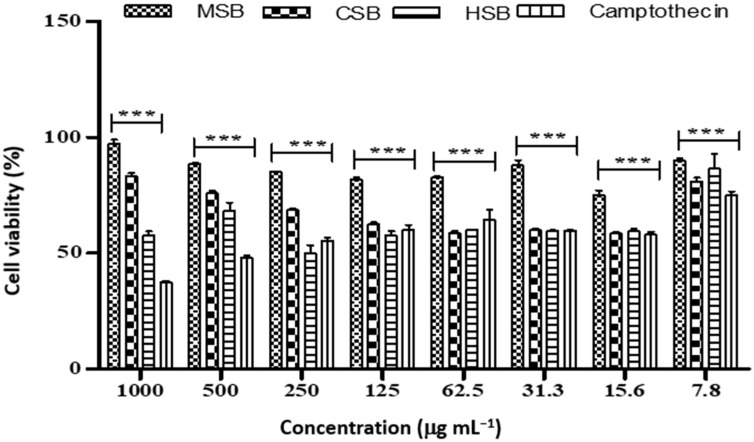
Cell viability of A549 cancer cell lines treated with different concentrations of *D. villosa* stem extract. *** (MSB vs. Camptothecin), *p* < 0.001.

In addition, the cell viability of A549 cells was observed to be lower on exposure to hexane stem extract (with IC_50_ value of 5.35 µg mL^−1^), chloroform stem extract (IC_50_ of 10.67 µg mL^−1^) and methanolic extract (13.48 µg mL^−1^) of *D. villosa* compared to control (IC_50_ of 19.26 µg mL^−1^) (Table 10 and Figure 8).

**Figure 9:**
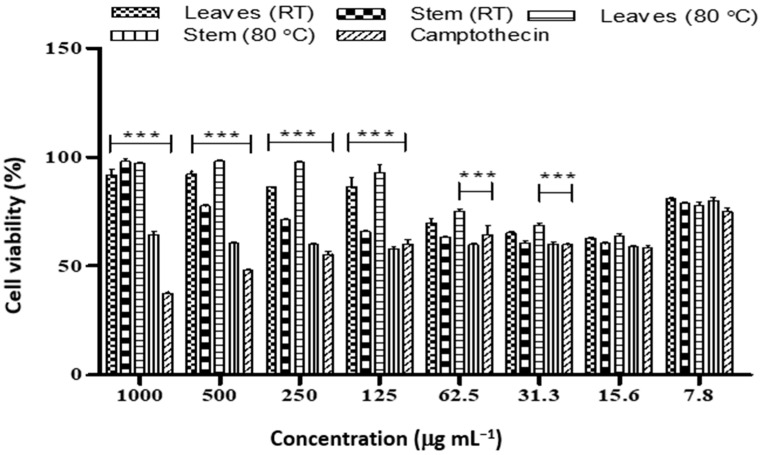
Cell viability of A549 cancer cell lines treated with different concentrations of nanoparticles synthesized from *D. villosa* leaves and stem extract at room temperature and 80 °C. *** (Leaves RT vs. Camptothecin), *p* < 0.001. *** (Leaves 80 °C vs. Camptothecin), *p* < 0.001.

The viability of A549 cells was observed to be lower on exposure to *D. villosa* stem nanoparticles at 80 °C and RT (IC_50_ values of 5.03 and 4.93 µg mL^−1^ respectively) compared to control (19.26 µg mL^−1^) (Table 11 and Figure 9).

## 3. Discussion

It was observed that the methanol extract produced maximum extraction yield. This is in line with Abdullah et al. [23] who reported previously that methanol extract of different plants usually yields significantly higher amounts compared to chloroform and hexane extract of same plants and it was further explained that it may be owing to occurrence of functional particles which are mostly polar organic phytochemical and are always available in most medicinal plants. For most studies, crude fibre, protein and good energy are considered as the main determinants of food types, and very few studies are available on the elemental composition of the *Diospyros* edible species. Crude fibre and protein in *Diospyros* leaves are well within the range as reported by earlier workers for other wild edibles [24,25]. The relatively high fibre content is an indication that the intake of *D. villosa* leaves could enhance peristalsis along gastrointestinal tract, digestion and even prevent constipation [26]. High fibre intake could lead to a reduced incidence of cohorts of metabolic syndrome disorder [27]. Dietary proteins are pivotal in the manufacturing and safeguarding of certain organic materials necessary for smooth functioning of human body [25]. The relatively high protein content of *D. villosa* could make it a useful supplement to diets with few proteins. Considering these nutritional values of *D. villosa*, the leaves seem to be fit for human consumption. However, there is still need for further identification and assessment of the protein make-up in the leaves and perhaps, the other vital and essential nutritive components of the plant.

*Diospyros villosa* leaves have long been recognized as a traditional medicinal plant. However, the putative anti-cancer effects of *D. villosa* leaves and their mechanisms of action have not been scientifically evaluated previously. As illustrated in Figure 1 and Figure 7, viability assays revealed that MCF-7 and A549 cells were more vulnerable to the plant extracts of *D. villosa* leaves than HEK293 cells. Similarly, a same trend was observed with *D. villosa* stem bark (Figure 2 and Figure 8). MCF-7 and A549 cells were more vulnerable to stem extracts of *D. villosa*. Thumbrain et al. [28] pinpointed that if an extract should be an anticancer agent, it should display toxicity on the A549 and MCF-7 cell lines while being somewhat less toxic to HEK293 cells. In this study, both leaves and stem bark exhibited strong cytotoxicity against MCF-7 and A549 cells but with disproportionate trend towards HEK293 cells. This shows that *D. villosa* extracts may not be toxic to normal cells, which makes it an ideal anticancer agent.

The exact mechanism of action through which the plant extracts exhibited its toxicity on cells was not established in this study. Earlier studies further demonstrated that most proteins in typical African diet come from high quality plant protein [29]. Elevated levels of protein and essential amino acids can inhibit the cancer progression and growth. This is in agreement with Gao et al. [30] who reported that plant proteins activated IGF-1 insulin signalling in order to regulate cancer growth and autophagy but to a very little extent. In addition, the presence of considerable amount of protein in the plant may be considered an avenue for building a complex compound with them embedded functional phytochemicals, some of which are thought to stop carcinogenesis through their antioxidant properties by interfering with oxidative stress signalling pathway and suppressing DNA damage. This is also in agreement with Kim et al. [31] where it was pointed out that a member of *Diospyros* genus (*Diospyros kaki*) exhibited cell death via activation of platelet-derived growth factor receptors (PDGFRs) which serve as the active binding site for membrane auto-phosphorylation. Our experimental findings support the notion that the incorporation of *D. villosa* protein into supplements may play a role in cancer inhibition and retrogression.

The results further showed that *D. villosa* leaves, stem and nanoparticles synthesized at room temperature were able to inhibit cell growth in vitro with high efficiency. Even more, the extracts showed more potency in MCF-7 cancer lines, displaying high cytotoxicity. This is in line with Park et al. [32] where *Diospyros kaki* (Thumb.) suppressed the proliferation of human cancer cell lines by decreasing cyclin D_1_ expression. Although, the mechanism of *D. villosa* cancer inhibition may not have been achieved through cyclin D_1_ expression, the excellent display of IC_50_ may be considered. In this present study, the *D. villosa* leaves, stem and biosynthesized nanoparticles with the highest anticancer activity presented IC_50_ showing potent inhibitory effect on the growth of MCF-7 and A549 cell lines. The results showed that the *D. villosa* plant presented the lower range of IC_50_ compared to a referenced anticancer medication, showing the higher potency. On the other hand, the methanolic extract of *D. villosa* plant showed the lowest potency in inhibiting cell growth in MCF-7 and A549 cells. Taking in account that the beneficial properties of *Diospyros* plants are related to a variety of bioactive components that enhance antioxidant capacity and consequently anticancer activity [33,34]. *Diospyros villosa* may be strong candidates for future cancer studies, having high antioxidant and anticancer activity. The activities of both hexane leaves and stem extracts were quite promising as effective anticancer agents. The hexane stem extract did not just only inhibit the growth of MCF-7 cells but also possess a lower IC_50_ compared to standard. The lowest value of IC_50_ as produced by methanolic leaves extracts would have been considered the best, but higher percentage viability of MCF-7 cells further explained that the methanolic leaves extract may rather be considered a strong antioxidant/antibacterial than anticancer agent. Also, the hexane stem extract displayed both lower IC_50_ value compared to the standard and a low percentage viability of A549 cells. In fact, the less toxicity of hexane leaves extract to HEK293 was observed as the IC_50_ values was quite higher. Both hexane stem extract and the synthesized stem nanoparticles at room temperature showed marked anticancer activities.

## 4. Materials and Methods

### 4.1. Plant Collection

Fresh samples of mature leaves and stem bark of *D. villosa* were collected from KwaZulu-Natal, Durban, South Africa (29°84′33.6″ S, 31°4′12″ E). The plant was identified and a voucher specimen was deposited in the Ward Herbarium (01/18257) at the School of Life Sciences, University of KwaZulu-Natal, Westville campus. The collected plant parts (leaves and stem bark) were washed, air-dried and pulverized into fine powder. The powdered samples were kept in a cool dry place for extraction purposes.

### 4.2. Plant Extraction

Powdered samples of the plant weighing 8 g were heated to a temperature of 40 °C for 15 min with 100 mL of 95% methanol in a round bottom flask attached to a Soxhlet apparatus. The crude extract was retained and the process was repeated thrice. Successive extractions using chloroform and hexane, respectively, were carried out after 30 min intervals. The condensate was further evaporated to dryness under reduced pressure at 40 °C in a rotary evaporator. The crude extract was stored at 4 °C and used within 48 h for further tests.

$$\mathrm{The}\;\mathrm{extraction}\;\mathrm{yield}\;(\%)=\frac{\mathrm{Weight}\;\mathrm{of}\;\mathrm{the}\;\mathrm{dry}\;\mathrm{extract}\;\left(g\right)}{\mathrm{Weight}\;\mathrm{of}\;\mathrm{the}\;\mathrm{sample}\;\mathrm{used}\;\mathrm{for}\;\mathrm{the}\;\mathrm{extraction}\;\left(g\right)}\;\times \;100.$$

### 4.3. Proximate Analysis

The proximate analysis of leaves and stem samples including the moisture, crude fibre, proteins, lipids, ash content and hydrolysable carbohydrates were assessed. Moisture content was determined by drying the leaves and stem samples at 80 °C in an oven until a constant weight was obtained [35]. The crude fibre was determined by the loss in weight on ignition of dried residue following the digestion of fat-free samples with 1.25% each of sulphuric acid and sodium hydroxide solutions [35]. The total protein content (N × 6.25) was estimated by the macro-Kjeldahl nitrogen assay method using a digestion apparatus combined with the photo-colourimetric method described by Baethgen and Alley [36]. The total lipids content was determined according to AOAC [37], by n-hexane extraction using an automatic Soxhlet analyzer (Soxtherm 2000 Automatic, C. Gerhardt, Northants, UK).

### 4.4. Synthesis of Silver Nanoparticles (AgNPs)

Silver nitrate (1 M AgNO_3_; Sigma Aldrich, South Africa) was prepared by dissolving 0.17 g in 100 mL of distilled water. Following this, 1 mM of AgNO_3_ was prepared by diluting 10 mL in distilled water (90 mL). The reduction of Ag^+^ was achieved by adding 5 mL of each *D. villosa* aqueous extract (leaves or stem bark) to 20 mL of 1 mM AgNO_3_. The mixtures were incubated for 24 h in the light at room temperature (RT; 24 °C). The procedure was repeated with the incubation at 80 °C by heating the extracts in a water bath for 60 min. The colour change from light yellow to dark brown was indicative of the presence of AgNPs [38,39]. Syntheses were performed in triplicate.

### 4.5. Quantification of AgNPs

Each AgNP solution was subjected to centrifugation using an Eppendorf microcentrifuge (5804/5804R, Sigma-Aldrich, Inc., St. Louis, MO, USA). The treatment solutions (leaves and stem bark at room temperature and 80 °C) were separately transferred into pre-weighed microcentrifuge tubes and purified for 2 h at 1650× *g* and 4 °C. The supernatant from each solution was decanted and the insoluble residue was reconstituted in 20 mL sterile distilled water and centrifuged repeatedly three more times for effective removal of unreacted materials. Samples were then oven-dried at 40 °C for 24 h after which the tubes were re-weighed to obtain the yield of the synthesized AgNPs.

### 4.6. Measurement of Cell Viability

Human embryonic kidney (HEK293), breast cancer (MCF-7) and human lung cancer (A549) cells were donated by the Department of Biotechnology, Durban University of Technology. Cells were grown at 37 °C in a humidified incubator under 5% CO_2_ in Dulbecco’s modified Eagle’s medium (DMEM) containing 10% Foetal Bovine Serum (FBS) and antibiotics (Penicillin; 10,000 U mL^−1^ and Streptomycin sulphate; 10,000 U mL^−1^ [Penicillin/Streptomycin]). Antibiotics change the phenotype and morphology of cells; therefore, the use of the antibiotics should be in very low concentrations, thus for this study, 1% Penicillin/Streptomycin was used. The cells were grown until 80% confluence was reached with media replaced as necessary. After confluence was reached, cells were trypsinized and sub-cultured. The 3-(4,5 dimethylthiazol-2-yl)-2,5-diphenyltetrazolium bromide (MTT) assay was used to determine the cytotoxicity of the extracts. The MTT assay was conducted according to Dwarka et al. [40] with minor modifications. Briefly, cells (50 μL of 1 × 10^2^ cells mL^−1^), as well as 50 μL of DMEM, were seeded into a 96-well flat bottom plate and incubated (37 °C for 24 h) in a humidified incubator under 5% CO_2_. Cells were then treated with 50 μL of extracts at varying concentrations (7.8–1000 μg mL^−1^) prepared in 5% DMSO and incubated for 24 h. Camptothecin was used as a positive control. MTT reagent (20 μL, 5 mg mL^−1^) was added to the cells and incubated at 37 °C for 4 h. One hundred microliters of DMSO was then added to each well to solubilise the formazan salt formed, and absorbance was read at 570 nm on a microplate spectrophotometer (Multiscan Go, Thermo Scientific, Waltham, MA, USA) for both treated and untreated cells. The percentage viability was calculated using the following formula:

$$\mathrm{Cell}\;\mathrm{viability}\;(\%)=\frac{\mathrm{Absorbance}\;\mathrm{of}\;\mathrm{treated}\;\mathrm{cells}}{\mathrm{Absorbance}\;\mathrm{of}\;\mathrm{untreated}\;\mathrm{cells}}\;\times \;100$$

### 4.7. Statistical Analysis

Data are displayed as means ± SEM. Statistical analysis was performed using Graph Pad Prism 5 (Graph Pad Software Inc., San Diego, CA, USA). The results were compared using one-way ANOVA followed by Bonferroni post hoc tests. Also, two-way ANOVA followed by Bonferroni post hoc tests was used where necessary. Effects were considered statistically significant at *p*-value < 0.05. The lower the half maximal inhibitory concentration (IC_50_) of the cancerous cell lines, the more the potency of the extract as an anticancer agent.

## 5. Conclusions

In this work, we have explored the nutritive contents and anticancer effect of the *D. villosa* leaves and stem bark as well as the nanoparticles against three cancer cell lines. The study revealed that *D. villosa* had high fibre and protein contents. *Diospyros villosa* may therefore be considered a plant with great potential as food supplement. It is further possible to conclude that *D. villosa* extracts potentially inhibited the viability of human breast carcinoma and lung carcinoma cells. Hence, its incorporation into nutritive supplements may provide a prophylactic regimen to both breast and lung cancer.

## Figures and Tables

**Table 1:** Yield of extracts of *D. villosa* leaves, stem bark and nanoparticles.

	Methanol (%)	Chloroform (%)	Hexane (%)	Nanoparticle at RT (g/g of Dry Plant Material)	Nanoparticle at 80 °C (g/g of Dry Plant Material)
Leaves	10.8	8.4	7.1	0.07	0.055
Stem bark	7.2	7.9	10.3	0.04	0.039

**Table 2:** Nutritional content (%) of investigated *D. villosa* leaves and stem bark.

					Literature	
	Emergent Leaves (%)	Young Leaves (%)	Mature Leaves (%)	Stem (%)	Leaves [22]	Stem [22]
Protein	8.50 ± 1.61	11.37 ± 0.68	14.95 ± 0.83 *	5.22 ± 0.72	11.49 ± 0.10	5.51 ± 0.10
Lipids	11.97 ± 1.36	11.39 ± 1.77	14.37 ± 0.16	13.34 ± 0.28	3.00 ± 0.01	1.83 ± 0.16
Crude fibre	29.73 ± 2.71 *	36.4 ± 3.49 *	29.60 ± 2.77 *	40.17 ± 3.63 *	2.66 ± 0.16	6.83 ± 0.33
Ash	8.33 ± 0.44	6.33 ± 0.60	6.67 ± 0.67	5.33 ± 0.60	11.16 ± 0.44	22.66 ± 0.33
Moisture	8.54 ± 0.75	9.67 ± 0.98	7.40 ± 0.80	2.13 ± 0.07	14.83 ± 0.44	11.33 ± 0.60
Carbohydrate	32.93 ± 0.62	24.84 ± 0.91	27.01 ± 0.44	33.81 ± 0.35	55.03 ± 0.01	50.96 ± 0.25

Values are mean ± SD of carefully conducted triplicate experiments. * *p* < 0.05.

**Table 3:** IC_50_ values of methanol, chloroform and hexane extract of *D. villosa* leaf against MCF-7 cell.

	Methanol Leaf Extr.	Chloroform Leaf Extr.	Hexane Leaf Extr.	Camptothecin
IC_50_	0.16	26.07	26.64	36.54

**Table 4:** IC_50_ values of methanol, chloroform and hexane extract of *D. villosa* stem against MCF-7 cell.

	Methanol Leaf Extr.	Chloroform Leaf Extr.	Hexane Leaf Extr.	Camptothecin
IC_50_	0.16	26.07	26.64	36.54

**Table 5:** IC_50_ values of *D. villosa* leaves and stem bark nanoparticles at both RT and 80 °C against MCF-7 cell.

	Leaves (RT)	Stem (RT)	Leaves (80 °C)	Stem (80 °C)	Camptothecin
IC_50_	2.03	4.08	2.53	5.11	36.54

**Table 6:** IC_50_ values of methanol, chloroform and hexane extract of *D. villosa* leaf against HEK293 cell.

	Methanol Leaf Extr.	Chloroform Leaf Extr.	Hexane Leaf Extr.	Camptothecin
IC_50_	158.5	198.5	41.85	14.77

**Table 7:** IC_50_ values of methanol, chloroform and hexane extract of *D. villosa* stem against HEK293 cell.

	Meth. Stem Extr.	Chloroform Stem Extr.	Hexane Stem Extr.	Camptothecin
IC_50_	45.1	3.93	0.10	14.77

**Table 8:** IC_50_ values of *D. villosa* leaves and stem bark nanoparticles at both RT and 80 °C against HEK293 cell.

	Leaves (RT)	Stem (RT)	Leaves (80 °C)	Stem (80 °C)	Camptothecin
IC_50_	4.77	7.09	333.80	51.36	14.77

**Table 9:** IC_50_ values of methanol, chloroform and hexane extract of *D. villosa* leaf against A549 cell.

	Methanol Leaf Extr.	Chloroform Leaf Extr.	Hexane Leaf Extr.	Camptothecin
IC_50_	7.76	4.59	7.09	19.26

**Table 10:** IC_50_ values of methanol, chloroform and hexane extract of *D. villosa* stem against A549 ceFll.

	Meth. Stem Extr.	Chloroform Stem Extr.	Hexane Stem Extr.	Camptothecin
IC_50_	10.67	5.35	13.48	19.26

**Table 11:** IC_50_ values of *D. villosa* leaves and stem bark nanoparticles at both RT and 80 °C against A549 cell.

	Leaves (RT)	Stem (RT)	Leaves (80 °C)	Stem (80 °C)	Camptothecin
IC_50_	7.13	4.93	33.80	5.03	19.26

## Data Availability

All data are presented in the article.
